# RNA Therapeutics Targeting Skeletal Muscle: Emerging Antisense and Gene-Modifying Strategies

**DOI:** 10.3390/biom16060794

**Published:** 2026-05-28

**Authors:** Takayuki Kuroda, Toshifumi Yokota

**Affiliations:** 1 Department of Medical Genetics, Faculty of Medicine and Dentistry, University of Alberta, Edmonton, AB T6G 2H7, Canada; tkuroda@ualberta.ca; 2 The Friends of Garrett Cumming Research & Muscular Dystrophy Canada, HM Toupin Neurological Sciences Research, Edmonton, AB T6G 2H7, Canada

**Keywords:** antisense oligonucleotide, phosphorodiamidate morpholino oligomer, small interfering RNA, clustered regularly interspaced short palindromic repeats and CRISPR-associated (CRISPR-Cas) system, cell-penetrating peptides, antibody–oligonucleotide conjugates, adeno-associated virus vector, Duchenne muscular dystrophy, myotonic dystrophy type 1, facioscapulohumeral muscular dystrophy

## Abstract

RNA-based therapeutics are reshaping the treatment landscape for skeletal muscle disorders by enabling modulation of RNA processing or direct correction of disease-causing alleles. In Duchenne muscular dystrophy (DMD), four antisense oligonucleotides—eteplirsen, golodirsen, viltolarsen, and casimersen—have received FDA approval; these phosphorodiamidate morpholino oligomers (PMOs) induce exon skipping to restore the reading frame and enable expression of internally truncated dystrophin. Beyond splice switching, RNA therapeutics include RNase H-active gapmers and steric-blocking antisense oligonucleotides (ASOs), small interfering RNAs (siRNAs) that mediate post-transcriptional gene silencing, and RNA-guided gene-modifying technologies such as CRISPR systems that can reframe or repair endogenous alleles. Despite major progress in DMD, broader clinical impact remains constrained by inefficient delivery to skeletal and especially cardiac muscle, the need for repeat administration for most modalities, and safety considerations that limit dose escalation and durability. Next-generation approaches aim to overcome these barriers through peptide- or antibody-conjugated oligonucleotides that enhance cellular uptake and tissue distribution, alternative chemistries with improved stability and potency, and viral or non-viral platforms for durable splice modulation. In parallel, CRISPR-based strategies—including base and prime editing—offer the prospect of one-time correction, while raising important questions regarding delivery, immunogenicity, editing specificity, and long-term safety. This review synthesizes recent advances in antisense and gene-modifying strategies for skeletal muscle and highlights practical priorities for translation, including improved muscle/heart delivery, controllable safety mechanisms, scalable manufacturing, and standardized biomarker-to-clinical outcome relationships.

## 1. Introduction to RNA-Based Therapeutics

RNA-based therapeutics comprise a broad spectrum of molecular mechanisms that enable precise modulation of gene expression at multiple levels [1]. These approaches range from antisense oligonucleotides that alter RNA processing or induce targeted degradation, to small interfering RNAs that silence transcripts through the RNA interference pathway, and RNA-guided editing systems that directly modify genomic DNA [2]. Although these modalities differ in their mechanisms, durability, and molecular targets, they collectively provide a versatile toolkit for correcting or compensating for pathogenic gene expression in skeletal muscle disorders [3,4].

### 1.1. Antisense Oligonucleotides (ASOs)

ASOs are single-stranded nucleic acids that bind to RNA and modulate its function [5]. The molecular mechanism of an ASO depends on its chemical modifications and the target RNA site. RNase H-dependent ASOs, also known as “gapmers,” bind to target mRNA and induce its degradation through RNase H, which specifically cleaves DNA/RNA duplexes [6]. Gapmer ASOs consist of a central DNA “gap” flanked by chemically modified nucleotides, typically 2′-O-methoxyethyl (2′-MOE) or locked nucleic acids (LNA), to increase binding affinity to the target RNA [7]. Gapmer ASOs also normally contain phosphorothioate (PS) internucleotide linkages instead of natural phosphodiester bonds to enhance nuclease stability [8]. However, PS linkages can contribute to toxicity through nonspecific protein binding, which remains a concern in clinical applications [9]. Another major class is RNase H-independent splice-switching oligonucleotides (SSOs), which bind to pre-mRNA and modulate splicing [10]. SSOs commonly incorporate 2′-MOE or other sugar modifications and typically include PS linkages for stability [11]. SSOs can also be synthesized using phosphorodiamidate morpholino oligomers (PMOs) [12]. PMOs are synthetic molecules designed to bind specific RNA sequences and block their processing or translation [13]. Unlike DNA or RNA, PMOs have an uncharged backbone, which confers high stability and resistance to nucleases. They only bind strongly to nearly perfectly complementary sequences. PMOs generally exhibit low innate immune activation and limited nonspecific protein binding; however, this can vary depending on sequence, dose, and conjugated ligand [14,15].

### 1.2. Small Interfering RNAs (siRNA)

siRNAs are short, double-stranded RNA molecules, typically 21–23 nucleotides in length [16]. They consist of a sense strand and an antisense strand. The antisense strand is incorporated into the RNA-induced silencing complex (RISC), which guides the complex to complementary target mRNA [17]. Within RISC, the core catalytic component is Argonaute-2 (AGO2), an endonuclease that cleaves the target mRNA through its “slicer” activity [18]. This AGO2-mediated cleavage generates site-specific mRNA degradation, leading to effective gene silencing through the RNA interference (RNAi) pathway [1]. Chemical modifications and delivery strategies are often used to enhance siRNA stability, reduce off-target effects, and improve cellular uptake [19].

### 1.3. RNA-Guided Gene-Modifying Technologies

RNA-guided gene-modifying technologies encompass both DNA-level genome editing and RNA-level transcript editing.

DNA-level genome editing approaches include the clustered regularly interspaced short palindromic repeats (CRISPR) and CRISPR-associated protein (CRISPR–Cas) systems, which introduce double-strand breaks (DSBs) at target DNA sequences complementary to a guide RNA [20,21]. Following DSB formation, cellular DNA repair pathways can modify the genome through non-homologous end joining (NHEJ) or homology-directed repair (HDR) [22]. In the presence of a donor DNA template, HDR enables the precise insertion or correction of genetic sequences [23]. More recently developed technologies, such as base editing and prime editing, utilize engineered CRISPR systems to introduce precise genetic modifications without generating DSBs [24]. Base editors enable single-nucleotide substitutions, whereas prime editors allow for targeted small insertions, deletions, or base substitutions with improved precision [25].

In contrast, RNA-level editing is mediated by adenosine deaminases acting on RNA (ADAR), endogenous enzymes that mediate adenosine-to-inosine (A-to-I) RNA editing within double-stranded RNA structures [26]. Inosine is interpreted as guanosine during translation, thereby altering the encoded protein sequence [27]. By harnessing this endogenous system, guide RNA-directed approaches have been developed to recruit ADAR to target mRNAs and induce site-specific A-to-I RNA editing [28].

### 1.4. Aptamers

Aptamers are short, structured single-stranded RNA or DNA oligonucleotides that bind target molecules with high affinity and specificity, functioning similarly to antibodies [29]. They are typically identified through the Systematic Evolution of Ligands by EXponential enrichment (SELEX) process, an iterative in vitro selection method that enriches oligonucleotides capable of binding a desired protein or receptor [30]. Because of their small size, chemical tunability, and low immunogenicity, aptamers are increasingly used as targeting ligands to enhance the cell-type specificity of RNA therapeutics, including siRNAs, ASOs, and oligonucleotide conjugates [31]. Recent studies have demonstrated aptamer-mediated delivery to muscle cells, highlighting their potential as modular components in next-generation RNA delivery platforms for skeletal muscle diseases [32].

### 1.5. MicroRNA (miRNA)-Based and Circular RNA (circRNA) Therapeutics

MicroRNA (miRNA) therapeutics include antisense inhibitors such as LNA-modified antimiRs (e.g., Miravirsen, which targets miR-122) and miRNA mimics that restore downregulated miRNA activity [33,34]. These approaches modulate post-transcriptional gene regulation and are being explored across metabolic, cardiovascular, and neuromuscular diseases [34]. Circular RNAs (circRNAs) have recently emerged as a stable RNA platform capable of long-lasting protein expression or regulatory functions, and synthetic circRNAs are now being developed as next-generation RNA therapeutics [35].

## 2. Delivery Strategies for RNA-Based Therapeutics

**Figure 1:**
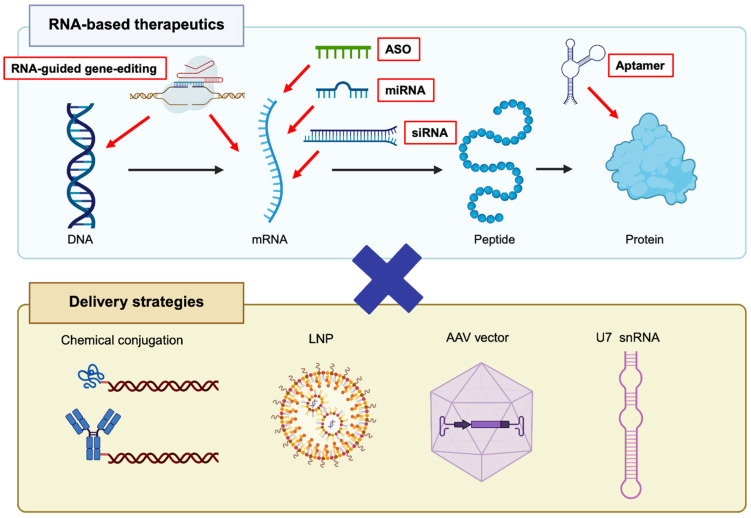
Overview of major RNA-based therapeutic modalities and delivery strategies. RNA therapeutics act at multiple levels of gene expression, including DNA- or RNA-targeting RNA-guided genome-editing systems, RNaseH-dependent mRNA degradation or pre-mRNA splicing modulation by antisense oligonucleotides (ASOs), post-transcriptional gene silencing mediated by small interfering RNAs (siRNAs) or microRNAs (miRNAs), and protein-level modulation through aptamer binding. These modalities can be paired with diverse delivery platforms—including chemical conjugation using peptide or antibody ligands, lipid nanoparticles (LNPs), adeno-associated virus (AAV) vectors, and U7 small nuclear RNA systems—to enhance cellular uptake, endosomal escape, nuclear access, and overall therapeutic activity. The figure was created with BioRender.com.

One of the main challenges for RNA-based therapeutics targeting skeletal muscle is efficient delivery [36]. Systemically administered RNA molecules are rapidly cleared from circulation and show limited accumulation in target tissues, reducing therapeutic efficacy [37]. To overcome this limitation, various drug delivery strategies have been developed [8] (Figure 1).

### 2.1. Chemical Conjugation

Chemical conjugation is a key approach to enhance delivery [15,38]. For example, N-acetylgalactosamine (GalNAc), an amino sugar derivative, acts as a ligand for the asialoglycoprotein receptor, enabling selective uptake of ASOs and siRNAs into hepatocytes [39]. Peptides and antibodies are also commonly used for conjugation. Another important strategy involves targeting transferrin receptor 1 (TfR1), a highly expressed iron-transport receptor on skeletal and cardiac muscle [40]. TfR1 undergoes receptor-mediated endocytosis and transcytosis, enabling efficient intracellular trafficking of ligand-bound cargo. This pathway has been leveraged for peptide- and antibody–oligonucleotide conjugates (AOCs) in which TfR1-binding antibodies or peptides facilitate tissue-specific delivery of ASOs and siRNAs to muscle [41]. Recent preclinical studies have demonstrated robust uptake and exon-skipping activity in skeletal muscle using TfR1-targeted AOCs, highlighting the receptor’s value as a delivery gateway for neuromuscular RNA therapeutics [42].

### 2.2. Lipid Nanoparticles (LNPs)

Lipid nanoparticles (LNPs) are nanoscale vesicles composed of four key components: ionizable lipids, helper lipids, cholesterol, and PEG-lipids, which together enable efficient encapsulation and delivery of nucleic acids [43]. After cellular uptake by endocytosis, protonation of ionizable lipids in the acidic endosome promotes membrane destabilization and release of the RNA cargo into the cytosol [43].

The clinical utility of LNPs was demonstrated by their use in COVID-19 mRNA vaccines, and LNPs are now considered one of the most promising platforms for RNA therapeutics [44,45]. Patisiran, an LNP-formulated siRNA targeting transthyretin (TTR) mRNA, became the first FDA-approved RNAi therapeutic, validating systemic LNP delivery in humans [46]. Recent engineering advances, including selective organ targeting (SORT) lipids, have broadened the applicability of LNPs to additional RNA modalities such as antisense oligonucleotides and guide RNAs [47].

### 2.3. Adeno-Associated Virus (AAV) Vectors

AAV vectors are widely used viral delivery systems that enable efficient transfer of genetic material into the nuclei of target cells, resulting in long-term transgene expression [48]. This sustained expression, often lasting for years, makes AAV vectors particularly suitable for therapeutic strategies requiring durable gene modulation [49]. In this context, RNA-guided gene-modifying technologies can be effectively delivered using AAV vectors by encoding the necessary components, such as guide RNAs and associated proteins, to enable long-term genome editing in target tissues [24]. Although extensively utilized in gene therapy applications, recent clinical experience with AAV vectors—particularly with high-dose systemic administration—has revealed toxicities including immune responses to the capsid, complement activation, hepatotoxicity, and dorsal root ganglion (DRG) toxicity [50,51,52].

### 2.4. U7 Small Nuclear RNA (snRNA)

snRNAs are components of small nuclear ribonucleoproteins (snRNPs), which are essential for pre-mRNA processing [53]. Among them, U7 snRNA is a specialized snRNA that functions in the 3′-end processing of replication-dependent histone pre-mRNAs [54]. Engineered U7 snRNA can be modified by replacing its natural histone-binding sequence and Sm-binding motif with antisense sequences targeting pre-mRNA [55]. In this form, U7 snRNP can modulate splicing by promoting exon skipping or inclusion, depending on the design of the antisense sequence. The main advantages of U7 snRNA-based approaches include its small genetic size, efficient nuclear localization, and generally low immunogenicity and cytotoxicity in cells [51,56].

### 2.5. Future Delivery Approaches Using Combinatorial Platforms

Next-generation delivery strategies aim to overcome the limitations of single-modality platforms. Antibody- and peptide-based conjugates provide enhanced tissue specificity, while AAV-delivered RNAi enables long-lasting gene silencing in post-mitotic tissues [57]. Cell-penetrating ligands and engineered receptor-binding peptides further improve intracellular trafficking and endosomal escape [41,58]. Combinatorial platforms—such as AOC-PMO, AOC-siRNA, and ligand-decorated LNPs—offer additive benefits by integrating the potency of oligonucleotides with the targeting precision of biologics, representing a promising direction for future RNA therapeutic development [41,42,59].

## 3. Disease Case Studies

The field of RNA therapeutics targeting skeletal muscle diseases are rapidly expanding [60]. In particular, for Duchenne muscular dystrophy (DMD), four antisense oligonucleotides—eteplirsen, golodirsen, viltolarsen, and casimersen—have received FDA approval [61]. For other skeletal muscle disorders, such as myotonic dystrophy type 1 (DM1) and facioscapulohumeral muscular dystrophy (FSHD), RNA-based therapeutics are currently under clinical investigation [41,62].

Despite these advances, several bottlenecks remain in clinical development, including limited therapeutic efficacy, delivery challenges, toxicity concerns, and the lack of robust clinical endpoints and biomarkers [63]. In addition, therapeutic efficacy often varies depending on tissue distribution and disease context [60]. This section summarizes the current status of RNA therapeutic development in skeletal muscle diseases and highlights emerging strategies to overcome these limitations.

### 3.1. Duchenne Muscular Dystrophy (DMD)

DMD is an X-linked recessive neuromuscular disorder that affects approximately 1 in 4000–5000 male births worldwide, with a global prevalence of ~3.6 per 100,000 individuals [64,65]. DMD is caused by mutations in the DMD gene, which encodes dystrophin, a key structural protein that stabilizes the sarcolemma during muscle contraction [66]. The absence of functional dystrophin leads to progressive muscle degeneration throughout the body, resulting in loss of ambulation typically by 10–12 years of age and premature mortality due to respiratory or cardiac failure [67]. Notably, cardiomyopathy remains a leading cause of death in patients with DMD [68].

**Figure 2:**
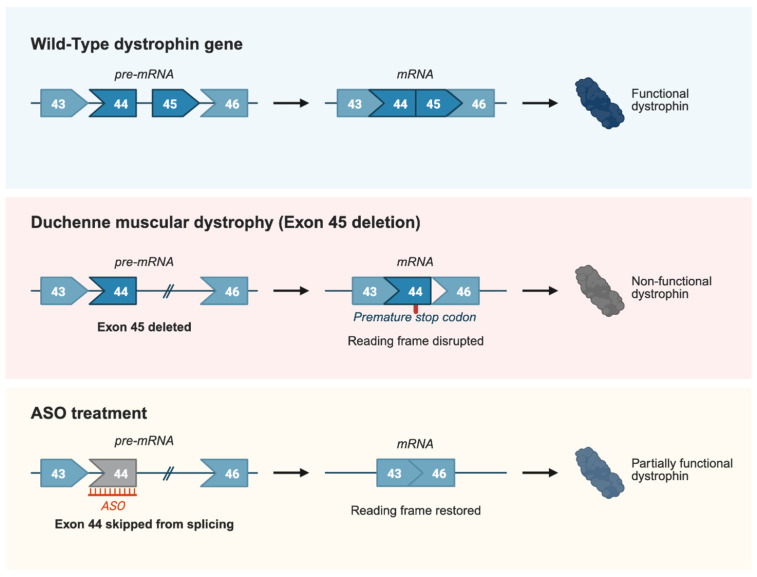
Splice-switching ASO restores the dystrophin reading frame in Duchenne muscular dystrophy. In the wild-type dystrophin gene, exons are spliced in-frame to produce full-length functional dystrophin. In DMD, deletion of exon 45 disrupts the reading frame, generating a premature stop codon and resulting in non-functional dystrophin. ASO-mediated exon 44 skipping removes exon 44, restores the reading frame, and enables production of a truncated but partially functional dystrophin protein. Figure was created with BioRender.com.

To date, more than 7000 distinct mutations in the DMD gene have been identified [69]. Mutation hotspots are located in exons 3–9 and 45–55 of the 79 exons, with deletions being the most common mutation [70]. These mutations frequently disrupt the reading frame, leading to premature termination codons and loss of functional dystrophin expression [71]. RNA-based therapeutics have emerged as promising approaches for DMD because they can directly target disease-causing mutations at the RNA level. In particular, antisense oligonucleotides (ASOs) targeting pre-mRNA can modulate splicing by inducing exon skipping, thereby restoring the reading frame and enabling the production of a truncated but partially functional dystrophin protein [72,73,74,75,76,77]. A schematic illustration of this mechanism is shown in Figure 2.

To date, four phosphorodiamidate morpholino oligomers (PMOs) have been approved by the FDA for DMD, all based on exon-skipping mechanisms but targeting different exons: eteplirsen (exon 51), golodirsen (exon 53), viltolarsen (exon 53), and casimersen (exon 45) [78,79,80,81]. These PMOs are administered as unconjugated (“naked”) oligonucleotides via weekly intravenous injection [61]. They act by sterically blocking splice sites on pre-mRNA, thereby inducing exon skipping to restore the reading frame and enable production of a truncated but partially functional dystrophin protein [82,83]. In clinical studies, these therapies have generally been well tolerated [84]. However, dystrophin restoration levels are typically low (often in the single-digit percentage range), and clinical benefits remain modest, with limited impact on disease progression [81,84]. One major limitation is inefficient delivery: due to their uncharged backbone, PMOs exhibit poor cellular uptake in skeletal muscle [85]. In particular, delivery to cardiac muscle is minimal, resulting in little or no therapeutic effect on cardiomyopathy, a leading cause of mortality in DMD patients [36].

Several strategies have been developed to enhance the therapeutic efficacy of PMOs in DMD. Dose escalation represents a straightforward approach; higher doses of eteplirsen (e.g., 100–200 mg/kg compared to earlier clinical doses of 30–50 mg/kg) are currently being evaluated for improved dystrophin restoration while assessing safety [64]. Another strategy involves the use of multiple PMOs targeting a single exon to enhance steric blockade of splicing regulatory elements and improve exon-skipping efficiency. For example, brogidirsen, a dual-targeting PMO designed for exon 44 skipping, is under phase 1/2 clinical evaluation and has demonstrated increased dystrophin expression, reaching approximately 10–20% in early studies (NCT05135663) [86].

Conjugation-based approaches have also shown promise. Peptide-conjugated PMOs (PPMOs) and antibody–oligonucleotide conjugates (AOC-PMOs), particularly those with antibodies against the transferrin receptor 1 (TfR1), enhance delivery to skeletal and cardiac muscle, addressing one of the major limitations of naked PMOs [42,87,88]. Several of these candidates are currently in clinical development. However, safety concerns remain, particularly for PPMOs [89]. For instance, SRP-5051 demonstrated hypomagnesemia associated with renal toxicity, leading to a temporary halt in clinical trials [14].

RNA-guided RNA-editing approaches have also been investigated for the treatment of DMD. Guide RNA (gRNA)-directed recruitment of ADAR has been shown to enable targeted RNA editing and partial restoration of the reading frame [90]. In particular, ADAR-mediated editing can convert a premature stop codon (e.g., UAG) into a sense codon (e.g., UGG), thereby enabling translation of a partially functional dystrophin protein [91]. This approach has been demonstrated as a proof-of-concept in the mdx mouse model of DMD [26]. Another study has reported that embedding guide RNAs into U7 snRNA scaffolds can enhance intracellular delivery, stability, and overall efficiency of ADAR-mediated RNA editing [92,93].

### 3.2. Myotonic Dystrophy Type 1 (DM1)

DM1 is an autosomal dominant, adult-onset neuromuscular disorder with an estimated global prevalence of approximately 1 in 8000 individuals [94]. DM1 is caused by an abnormal expansion of CTG trinucleotide repeats in the 3′ untranslated region of the DMPK gene, leading to the production of toxic CUG-repeat-containing RNA transcripts [95]. These mutant RNAs sequester RNA-binding proteins, particularly muscleblind-like (MBNL) proteins, resulting in widespread dysregulation of alternative splicing across multiple tissues [96,97].

Clinically, DM1 is a multisystem disorder characterized by progressive skeletal muscle weakness, cardiac conduction abnormalities, respiratory insufficiency, endocrine dysfunction, cognitive and behavioral impairments, and early-onset cataracts [98]. Among these manifestations, cardiac arrhythmias and conduction defects are major contributors to morbidity and mortality [99]. Currently, no disease-modifying therapies have been approved for DM1.

Clinical development of RNA-based therapeutics targeting the DMPK gene is ongoing. The first antisense oligonucleotide (ASO), the gapmer baliforsen, was well tolerated in patients with DM1; however, it failed to achieve sufficient concentrations in skeletal muscle, highlighting delivery as a major limitation [100,101]. To address this challenge, next-generation delivery approaches are being explored. Antibody–oligonucleotide conjugates (AOCs), particularly transferrin receptor 1 (TfR1)-targeting gapmer ASOs (ACHIEVE trial, NCT05481879), are currently under clinical evaluation. In addition, TfR1-targeting siRNA conjugates have been assessed in clinical trials (MARINA trial, NCT05027269), demonstrating reductions in mutant DMPK mRNA levels, increased availability of functional MBNL proteins, and partial correction of disease-associated mis-splicing in a dose-dependent manner, with trends toward improved muscle function [62].

CRISPR–Cas9-based approaches have also been investigated as therapeutic strategies for DM1 by directly targeting the expanded CTG repeats in the DMPK gene [102,103]. For example, in transgenic mouse models, delivery of CRISPR–Cas9 via adeno-associated virus (AAV) vectors, together with two single guide RNAs (sgRNAs) flanking the repeat region, enables excision of the expanded CTG tract [104]. This results in improvements in both molecular and functional phenotypes in skeletal and cardiac muscle [104]. In addition to repeat excision, alternative strategies have been explored to suppress DMPK expression. Targeting the DMPK promoter using CRISPR-based approaches has been reported to reduce transcription by up to 80% [105]. Although these genome-editing modalities show considerable promise, their clinical translation remains at an early stage, and further studies are required to establish their safety, delivery efficiency, and long-term effects in patients [106].

Aptamer-based targeting strategies are also being explored to enhance the delivery of RNA therapeutics to skeletal muscle [107]. Muscle-homing RNA aptamers have been shown to increase the uptake of siRNAs and antisense oligonucleotides in muscle cells, demonstrating their potential as modular ligands for precision delivery in DMD models [32,108].

### 3.3. Facioscapulohumeral Muscular Dystrophy (FSHD)

FSHD is an autosomal dominant, adult-onset muscular disorder and one of the most common forms of muscular dystrophy, affecting an estimated 45,000–87,000 individuals in the United States and Europe [109]. FSHD is caused by aberrant expression of the transcription factor double homeobox 4 (DUX4), which activates a cascade of germline and pro-apoptotic genes, leading to toxicity in skeletal muscle [110,111]. In healthy individuals, DUX4 expression is epigenetically silenced. In FSHD type 1, contraction of the D4Z4 macrosatellite repeat array, and in FSHD type 2, mutations in epigenetic regulators such as structural maintenance of chromosomes flexible hinge domain containing 1 (SMCHD1), lead to chromatin relaxation and inappropriate DUX4 expression [112]. Although disease onset typically occurs in adolescence or early adulthood with progressive weakness of facial, scapular, and humeral muscles, approximately 7–15% of patients present in childhood with a more severe phenotype, often accompanied by hearing loss and retinal vasculopathy [113]. Currently, no disease-modifying therapies have been approved.

Antisense oligonucleotides (ASOs) targeting DUX4 mRNA have demonstrated preclinical efficacy. Gapmer ASOs have shown robust suppression of DUX4 expression in patient-derived FSHD myotubes and mouse models [114,115]. PMOs have also been reported to reduce DUX4 expression by sterically blocking the polyadenylation signal or coding regions of the transcript, resulting in improved pathological features in animal models [116].

To enhance delivery, antibody–oligonucleotide conjugate (AOC) strategies, particularly transferrin receptor 1 (TfR1)-targeted antibody–siRNA conjugates such as AOC 1020, have been investigated [109]. This approach reduced DUX4-regulated gene expression by approximately 75% in patient-derived myotubes and prevented muscle weakness and fibrosis following a single systemic dose in mouse models [109]. The safety and efficacy of these strategies are currently being evaluated in clinical trials.

## 4. Translational Priorities

RNA-based therapeutics for skeletal muscle disorders share several cross-cutting translational challenges that influence their clinical development, regardless of modality (ASO, siRNA, RNA editing), delivery strategy (peptide-conjugate, AOC, LNP, AAV, U7), or disease target (DMD, DM1, FSHD) [117]. These include the selection of predictive animal models, the establishment of robust biomarkers and clinical endpoints, the management of safety liabilities, and the development of scalable manufacturing and regulatory frameworks [118]. Addressing these priorities is essential for advancing RNA therapeutics from preclinical proof-of-concept to meaningful and durable clinical benefit.

### 4.1. Animal Models

In addition to cell models, rodent models have been indispensable for mechanistic studies and early proof-of-concept studies for the treatment of skeletal muscle diseases [119]. However, their ability to predict human responses remains limited. For example, mdx mice exhibit mild pathology, rapid metabolism, and efficient muscle regeneration, which obscure long-term degenerative trajectories [120]. Moreover, systemic delivery of ASOs or siRNAs in mice does not accurately reflect human biodistribution challenges, particularly for cardiac and diaphragm uptake [119]. These discrepancies often lead to overestimation of therapeutic efficacy and underestimation of toxicity [121].

Large-animal models provide an essential translational bridge for RNA therapeutics. Canine DMD models (GRMD, CXMDJ) closely recapitulate human disease progression—including muscle weakness, respiratory decline, and cardiomyopathy—and have been widely used to evaluate exon-skipping ASOs and gene therapies [122,123]. Complementing canine models, the genome-edited microminipig with DMD exon 23 mutation (DMD-MMP) exhibits early locomotor deficits, progressive myocardial fibrosis, and declining left-ventricular ejection fraction, enabling longitudinal assessment of systemic dosing, imaging, and tissue biopsies [124]. Together, dogs and microminipigs provide complementary strengths for evaluating delivery, biodistribution, and chronic safety [125]. Beyond DMD, expanding large-animal resources—including non-human primates (NHPs)—is critical for assessing the pharmacokinetics, immunogenicity, and tissue distribution of oligonucleotide therapeutics [126]. NHPs offer the closest approximation to human receptor biology and immune responses, making them indispensable for evaluating advanced modalities such as AOCs, LNPs, and AAV vectors [109,127].

### 4.2. Endpoints and Biomarkers

The development of sensitive and disease-relevant biomarkers is essential for translating RNA-based therapeutics in skeletal muscle disorders [128,129]. Molecular biomarkers such as exon-skipping efficiency, target transcript reduction, and correction of disease-related RNA splicing or transcriptional abnormalities provide direct measures of target engagement and RNA repair [130]. Quantification of dystrophin or other muscle proteins by Western blot or mass spectrometry remains a regulatory-accepted pharmacodynamic endpoint in DMD [131]. Circulating biomarkers, including serum creatine kinase and cardiac troponins, are widely used, while emerging markers such as myomiRs (miR-1, miR-133, miR-206) offer non-invasive readouts of muscle injury and regeneration [132]. Imaging biomarkers, particularly quantitative MRI, enable sensitive detection of muscle degeneration and fibrosis and are increasingly incorporated into clinical trials [133]. Functional endpoints such as the 6-min walk test, motor function measure (MFM), North Star Ambulatory Assessment (NSAA) for DMD, myotonia/respiratory assessments for DM1, reachable workspace for FSHD, and the Hammersmith Functional Motor Scale (HFMS) for spinal muscular atrophy remain essential for linking molecular effects to clinical benefit [134,135,136,137,138]. Standardization across trials and harmonization of biomarker panels will be critical for regulatory acceptance.

### 4.3. Safety Considerations

Safety remains a central determinant of clinical translation for RNA therapeutics [139]. PMOs are generally well tolerated but require high systemic doses due to limited tissue uptake [78]. PPMOs improve delivery but have shown renal toxicity and hypomagnesemia in clinical studies, highlighting the need for careful monitoring [14]. Gapmer ASOs and siRNAs carry risks of unintended off-target gene silencing or toxicity related to protein interactions [9,140]. Delivery platforms introduce additional risks: AOCs may induce immunogenicity or receptor saturation; LNPs can induce complement activation or infusion reactions; and AAV vectors raise concerns regarding long-term expression, integration, and immune responses [44,51,141]. In addition to these platform-specific toxicities, immune activation remains a major challenge for oligonucleotide therapeutics, particularly for unmethylated motifs or double-stranded RNA structures that can stimulate innate immune sensors [142]. Allele specificity is another critical safety consideration for dominant-negative or toxic-gain-of-function disorders, where incomplete discrimination between mutant and wild-type alleles may lead to unintended loss of essential gene function [143]. Because skeletal muscle diseases require chronic administration over years, long-term safety—particularly in cardiac and diaphragm tissues—must be evaluated in predictive large-animal models such as canine DMD and microminipigs [122,125].

### 4.4. Manufacturing and Regulatory Considerations

Manufacturing RNA therapeutics at clinical scale requires stringent control of chemical purity, stereochemistry, backbone integrity, and impurity profiles, particularly for PMOs, gapmers, and siRNAs [144]. Conjugated modalities such as AOCs and PPMOs demand reproducible linker chemistry, with AOCs requiring consistent drug–antibody ratios and PPMOs requiring control of peptide-oligonucleotide conjugation stoichiometry, complicating scale-up [41]. Regulatory agencies increasingly require mechanistic justification, robust biodistribution and toxicology data in two species, and standardized assays for target RNA or protein modulation [145]. Off-target assessment using genomic profiling and RNA sequencing has become increasingly essential for evaluating the specificity and safety of RNA-based therapeutics [146,147]. Chemistry, Manufacturing, and Controls (CMC) frameworks must ensure identity, potency, stability, and batch-to-batch consistency [148]. As hybrid platforms (AOC-PMO, AOC-siRNA, AAV-RNA editors) expand, regulatory pathways must adapt to accommodate their distinct pharmacology and safety profiles.

## 5. Conclusions

RNA-based therapeutics have emerged as a clinically validated strategy for treating skeletal muscle disorders, supported by advances in RNA degradation, exon skipping, RNA interference, and RNA-editing technologies. Yet their successful translation requires bridging the gap between molecular efficacy and clinically meaningful outcomes. Rodent models remain indispensable for mechanistic discovery, but large-animal systems such as canine DMD models and genome-edited microminipigs provide more predictive platforms for evaluating delivery, biodistribution, and long-term safety. Progress in sensitive biomarkers, quantitative imaging, and standardized functional assessments is strengthening the ability to measure target engagement and therapeutic benefit. At the same time, careful attention to off-target effects, delivery-related toxicities, and evolving manufacturing and regulatory expectations is essential. Together, these advances are laying the foundation for safer, more effective, and more durable RNA-based therapies that can ultimately transform care for patients with neuromuscular disease.

## Data Availability

No new data were created or analyzed in this study. Data sharing is not applicable to this article.

## References

[1] Sparmann A., Vogel J. (2023). RNA-based Medicine: From Molecular Mechanisms to Therapy. EMBO J..

[2] Miao Y., Fu C., Yu Z., Yu L., Tang Y., Wei M. (2024). Current Status and Trends in Small Nucleic Acid Drug Development: Leading the Future. Acta Pharm. Sin. B.

[3] Egli M., Manoharan M. (2023). Chemistry, Structure and Function of Approved Oligonucleotide Therapeutics. Nucleic Acids Res..

[4] Andrea Z.A., Matteo F.Y., Alessandra B., Carlo P.S. (2024). Molecular Mechanisms and Therapeutic Strategies for Neuromuscular Diseases. Cell. Mol. Life Sci..

[5] Bennett C.F., Krainer A.R., Cleveland D.W. (2019). Antisense Oligonucleotide Therapies for Neurodegenerative Diseases. Annu. Rev. Neurosci..

[6] Sardone V., Zhou H., Muntoni F., Ferlini A., Falzarano M.S. (2017). Antisense Oligonucleotide-Based Therapy for Neuromuscular Disease. Molecules.

[7] Crooke S.T., Liang X.H., Baker B.F., Crooke R.M. (2021). Antisense Technology: A Review. J. Biol. Chem..

[8] Eckstein F. (2014). Phosphorothioates, Essential Components of Therapeutic Oligonucleotides. Nucleic Acid Ther..

[9] Shen W., De Hoyos C.L., Migawa M.T., Vickers T.A., Sun H., Low A., Bell T.A., Rahdar M., Mukhopadhyay S., Hart C.E. (2019). Chemical Modification of PS-ASO Therapeutics Reduces Cellular Protein-Binding and Improves the Therapeutic Index. Nat. Biotechnol..

[10] Havens M.A., Hastings M.L. (2016). Splice-Switching Antisense Oligonucleotides as Therapeutic Drugs. Nucleic Acids Res..

[11] Wilton S.D., Fletcher S. (2011). RNA splicing manipulation: Strategies to modify gene expression for a variety of therapeutic outcomes. Curr. Gene Ther..

[12] Zhu A., Chiba S., Shimizu Y., Kunitake K., Okuno Y., Aoki Y., Yokota T. (2023). Ensemble-Learning and Feature Selection Techniques for Enhanced Antisense Oligonucleotide Efficacy Prediction in Exon Skipping. Pharmaceutics.

[13] Aslesh T., Maruyama R., Yokota T. (2018). Skipping Multiple Exons to Treat DMD-Promises and Challenges. Biomedicines.

[14] Sheikh O., Yokota T. (2022). Pharmacology and Toxicology of Eteplirsen and SRP-5051 for DMD Exon 51 Skipping: An Update. Arch. Toxicol..

[15] Sabrina Haque U., Kohut M., Yokota T. (2024). Comprehensive Review of Adverse Reactions and Toxicology in ASO-Based Therapies for Duchenne Muscular Dystrophy: From FDA-Approved Drugs to Peptide-Conjugated ASO. Curr. Res. Toxicol..

[16] Ebenezer O., Oyebamiji A.K., Olanlokun J.O., Tuszynski J.A., Wong G.K.S. (2025). Recent Update on SiRNA Therapeutics. Int. J. Mol. Sci..

[17] Iwakawa H.-O., Tomari Y. (2022). Life of RISC: Formation, Action, and Degradation of RNA-Induced Silencing Complex. Mol. Cell.

[18] Liu J., Carmell M.A., Rivas F.V., Marsden C.G., Thomson J.M., Song J.J., Hammond S.M., Joshua-Tor L., Hannon G.J. (2004). Argonaute2 Is the Catalytic Engine of Mammalian RNAi. Science.

[19] Moazzam M., Zhang M., Hussain A., Yu X., Huang J., Huang Y. (2024). The Landscape of Nanoparticle-Based SiRNA Delivery and Therapeutic Development. Mol. Ther..

[20] Jinek M., Chylinski K., Fonfara I., Hauer M., Doudna J.A., Charpentier E. (2012). A Programmable Dual-RNA-Guided DNA Endonuclease in Adaptive Bacterial Immunity. Science.

[21] Park S.J., Lee G.E., Cho S.M., Choi E.H. (2025). Recent Applications, Future Perspectives, and Limitations of the CRISPR-Cas System. Mol. Ther. Nucleic Acids.

[22] Lieber M.R. (2008). The Mechanism of Human Nonhomologous DNA End Joining. J. Biol. Chem..

[23] Mali P., Yang L., Esvelt K.M., Aach J., Guell M., DiCarlo J.E., Norville J.E., Church G.M. (2013). RNA-Guided Human Genome Engineering via Cas9. Science.

[24] Kantor A., McClements M.E., Maclaren R.E. (2020). Crispr-Cas9 Dna Base-Editing and Prime-Editing. Int. J. Mol. Sci..

[25] Anzalone A.V., Koblan L.W., Liu D.R. (2020). Genome Editing with CRISPR–Cas Nucleases, Base Editors, Transposases and Prime Editors. Nat. Biotechnol..

[26] Katrekar D., Chen G., Meluzzi D., Ganesh A., Worlikar A., Shih Y.R., Varghese S., Mali P. (2019). In Vivo RNA Editing of Point Mutations via RNA-Guided Adenosine Deaminases. Nat. Methods.

[27] Cohen-Fultheim R., Twersky I., Krupkin H., Roth S.H., Levanon E.Y., Eisenberg E. (2025). A Cytoplasmic Index for Quantifying Immune-Related A-to-I RNA Editing. bioRxiv.

[28] Booth B.J., Nourreddine S., Katrekar D., Savva Y., Bose D., Long T.J., Huss D.J., Mali P. (2023). RNA Editing: Expanding the Potential of RNA Therapeutics. Mol. Ther..

[29] Ellington A.D., Szostak J.W. (1990). In Vitro Selection of RNA Molecules That Bind Specific Ligands. Nature.

[30] Tuerk C., Gold L. (1990). Systematic Evolution of Ligands by Exponential Enrichment: RNA Ligands to Bacteriophage T4 DNA Polymerase. Science.

[31] Keefe A.D., Pai S., Ellington A. (2010). Aptamers as Therapeutics. Nat. Rev. Drug Discov..

[32] Millozzi F., Milán-Rois P., Sett A., Delli Carpini G., De Bardi M., Gisbert-Garzarán M., Sandonà M., Rodríguez-Díaz C., Martínez-Mingo M., Pardo I. (2025). Aptamer-Conjugated Gold Nanoparticles Enable Oligonucleotide Delivery into Muscle Stem Cells to Promote Regeneration of Dystrophic Muscles. Nat. Commun..

[33] Janssen H.L.A., Reesink H.W., Lawitz E.J., Zeuzem S., Rodriguez-Torres M., Patel K., van der Meer A.J., Patick A.K., Chen A., Zhou Y. (2013). Treatment of HCV Infection by Targeting MicroRNA. N. Engl. J. Med..

[34] van Rooij E., Kauppinen S. (2014). Development of Micro RNA Therapeutics Is Coming of Age. EMBO Mol. Med..

[35] Wesselhoeft R.A., Kowalski P.S., Anderson D.G. (2018). Engineering Circular RNA for Potent and Stable Translation in Eukaryotic Cells. Nat. Commun..

[36] Echigoya Y., Nakamura A., Nagata T., Urasawa N., Lim K.R.Q., Trieu N., Panesar D., Kuraoka M., Moulton H.M., Saito T. (2017). Effects of Systemic Multiexon Skipping with Peptide-Conjugated Morpholinos in the Heart of a Dog Model of Duchenne Muscular Dystrophy. Proc. Natl. Acad. Sci. USA.

[37] Paunovska K., Loughrey D., Dahlman J.E. (2022). Drug Delivery Systems for RNA Therapeutics. Nat. Rev. Genet..

[38] Debacker A.J., Voutila J., Catley M., Blakey D., Habib N. (2020). Delivery of Oligonucleotides to the Liver with GalNAc: From Research to Registered Therapeutic Drug. Mol. Ther..

[39] Springer A.D., Dowdy S.F. (2018). GalNAc-SiRNA Conjugates: Leading the Way for Delivery of RNAi Therapeutics. Nucleic Acid Ther..

[40] Li H., Qian Z.M. (2002). Transferrin/Transferrin Receptor-Mediated Drug Delivery. Med. Res. Rev..

[41] Malecova B., Burke R.S., Cochran M., Hood M.D., Johns R., Kovach P.R., Doppalapudi V.R., Erdogan G., Arias J.D., Darimont B. (2023). Targeted Tissue Delivery of RNA Therapeutics Using Antibody-Oligonucleotide Conjugates (AOCs). Nucleic Acids Res..

[42] Etxaniz U., Marks I., Albin T., Diaz M., Bhardwaj R., Anderson A., Tyaglo O., Hoang T., Missinato M.A., Svensson K. (2025). AOC 1044 Induces Exon 44 Skipping and Restores Dystrophin Protein in Preclinical Models of Duchenne Muscular Dystrophy. Nucleic Acids Res..

[43] Cullis P.R., Felgner P.L. (2024). The 60-Year Evolution of Lipid Nanoparticles for Nucleic Acid Delivery. Nat. Rev. Drug Discov..

[44] Dézsi L., Mészáros T., Kozma G., H-Velkei M., Oláh C.Z., Szabó M., Patkó Z., Fülöp T., Hennies M., Szebeni M. (2022). A Naturally Hypersensitive Porcine Model May Help Understand the Mechanism of COVID-19 MRNA Vaccine-Induced Rare (Pseudo) Allergic Reactions: Complement Activation as a Possible Contributing Factor. Geroscience.

[45] Schoenmaker L., Witzigmann D., Kulkarni J.A., Verbeke R., Kersten G., Jiskoot W., Crommelin D.J.A. (2021). MRNA-Lipid Nanoparticle COVID-19 Vaccines: Structure and Stability. Int. J. Pharm..

[46] Adams D., Gonzalez-Duarte A., O’Riordan W.D., Yang C.-C., Ueda M., Kristen A.V., Tournev I., Schmidt H.H., Coelho T., Berk J.L. (2018). Patisiran, an RNAi Therapeutic, for Hereditary Transthyretin Amyloidosis. N. Engl. J. Med..

[47] Roy Chowdhury C., Hoover E.C., Day E.S. (2025). Membrane-Modified Lipid Nanoparticles for RNA Delivery. Mol. Ther. Methods Clin. Dev..

[48] Pozdniakova N., Generalov E., Shevelev A., Tarasova O. (2025). RNA Therapeutics: Delivery Problems and Solutions—A Review. Pharmaceutics.

[49] Wang J.H., Gessler D.J., Zhan W., Gallagher T.L., Gao G. (2024). Adeno-Associated Virus as a Delivery Vector for Gene Therapy of Human Diseases. Signal Transduct. Target. Ther..

[50] Hawley Z.C.E., Pardo I.D., Cao S., Zavodszky M.I., Casey F., Ferber K., Luo Y., Hana S., Chen S.K., Doherty J. (2025). Dorsal Root Ganglion Toxicity after AAV Intra-CSF Delivery of a RNAi Expression Construct into Non-Human Primates and Mice. Mol. Ther..

[51] Mingozzi F., High K.A. (2013). Immune Responses to AAV Vectors: Overcoming Barriers to Successful Gene Therapy. Blood.

[52] Hordeaux J., Lamontagne R.J., Song C., Buchlis G., Dyer C., Buza E.L., Ramezani A., Wielechowski E., Greig J.A., Chichester J.A. (2024). High-Dose Systemic Adeno-Associated Virus Vector Administration Causes Liver and Sinusoidal Endothelial Cell Injury. Mol. Ther..

[53] Gadgil A., Raczyńska K.D. (2021). U7 SnRNA: A Tool for Gene Therapy. J. Gene Med..

[54] Ideue T., Adachi S., Naganuma T., Tanigawa A., Natsume T., Hirose T. (2012). U7 Small Nuclear Ribonucleoprotein Represses Histone Gene Transcription in Cell Cycle-Arrested Cells. Proc. Natl. Acad. Sci. USA.

[55] Lesman D., Rodriguez Y., Rajakumar D., Wein N. (2021). U7 SnRNA, a Small RNA with a Big Impact in Gene Therapy. Hum. Gene Ther..

[56] Vulin A., Barthélémy I., Goyenvalle A., Thibaud J.L., Beley C., Griffith G., Benchaouir R., Le Hir M., Unterfinger Y., Lorain S. (2012). Muscle Function Recovery in Golden Retriever Muscular Dystrophy after AAV1-U7 Exon Skipping. Mol. Ther..

[57] Bisset D.R., Stepniak-Konieczna E.A., Zavaljevski M., Wei J., Carter G.T., Weiss M.D., Chamberlain J.R. (2015). Therapeutic Impact of Systemic AAV-Mediated RNA Interference in a Mouse Model of Myotonic Dystrophy. Hum. Mol. Genet..

[58] McClorey G., Banerjee S. (2018). Cell-Penetrating Peptides to Enhance Delivery of Oligonucleotide-Based Therapeutics. Biomedicines.

[59] Lin Y., Cheng Q., Wei T. (2023). Surface Engineering of Lipid Nanoparticles: Targeted Nucleic Acid Delivery and Beyond. Biophys. Rep..

[60] Bubenik J.L., Scotti M.M., Swanson M.S. (2024). Therapeutic Targeting of RNA for Neurological and Neuromuscular Disease. Genes Dev..

[61] Eser G., Topaloğlu H. (2022). Current Outline of Exon Skipping Trials in Duchenne Muscular Dystrophy. Genes.

[62] Kwan T.T., Meng Q., Delos Santos N., Johnson N.E., Thornton C.A., Malecova B., Burke R., Tai L.J., Levin A.A., Younis H.S. (2026). *Delpacibart etedesiran* Improves the Molecular Pathology of Myotonic Dystrophy Type 1 in the Phase 1/2 MARINA Study. Mol. Ther..

[63] De Serres-Bérard T., Ait Benichou S., Jauvin D., Boutjdir M., Puymirat J., Chahine M. (2022). Recent Progress and Challenges in the Development of Antisense Therapies for Myotonic Dystrophy Type 1. Int. J. Mol. Sci..

[64] Chassin A., Ono H., Ashida Y., Imamura M., Aoki Y. (2026). RNA Therapeutics for Duchenne Muscular Dystrophy: Exon Skipping, RNA Editing, and Translational Insights from Genome-Edited Microminipig Models. Int. J. Mol. Sci..

[65] Salari N., Fatahi B., Valipour E., Kazeminia M., Fatahian R., Kiaei A., Shohaimi S., Mohammadi M. (2022). Global Prevalence of Duchenne and Becker Muscular Dystrophy: A Systematic Review and Meta-Analysis. J. Orthop. Surg. Res..

[66] Hoffman E.P., Brown R.H., Kunkel L.M. (1987). Dystrophin: The Protein Product of the Duchenne Muscular Dystrophy Locus. Cell.

[67] Messina S., Vita G.L. (2018). Clinical Management of Duchenne Muscular Dystrophy: The State of the Art. Neurol. Sci..

[68] D’Ambrosio E.S., Mendell J.R. (2023). Evolving Therapeutic Options for the Treatment of Duchenne Muscular Dystrophy. Neurotherapeutics.

[69] Bladen C.L., Salgado D., Monges S., Foncuberta M.E., Kekou K., Kosma K., Dawkins H., Lamont L., Roy A.J., Chamova T. (2015). The TREAT-NMD DMD Global Database: Analysis of More than 7,000 Duchenne Muscular Dystrophy Mutations. Hum. Mutat..

[70] Leckie J., Zia A., Yokota T. (2024). An Updated Analysis of Exon-Skipping Applicability for Duchenne Muscular Dystrophy Using the UMD-DMD Database. Genes.

[71] Bez Batti Angulski A., Hosny N., Cohen H., Martin A.A., Hahn D., Bauer J., Metzger J.M. (2023). Duchenne Muscular Dystrophy: Disease Mechanism and Therapeutic Strategies. Front. Physiol..

[72] Takeda S., Clemens P.R., Hoffman E.P. (2021). Exon-Skipping in Duchenne Muscular Dystrophy. J. Neuromuscul. Dis..

[73] Dunckley M.G., Manoharan M., Villiet P., Eperon I.C., Dickson G. (1998). Modification of Splicing in the Dystrophin Gene in Cultured Mdx Muscle Cells by Antisense Oligoribonucleotides. Hum. Mol. Genet..

[74] Mann C.J., Honeyman K., Cheng A.J., Ly T., Lloyd F., Fletcher S., Morgan J.E., Partridge T.A., Wilton S.D. (2001). Antisense-Induced Exon Skipping and Synthesis of Dystrophin in the Mdx Mouse. Proc. Natl. Acad. Sci. USA.

[75] Aartsma-Rus A., Janson A.A.M., Kaman W.E., Bremmer-Bout M., den Dunnen J.T., Baas F., van Ommen G.J.B., van Deutekom J.C.T. (2003). Therapeutic Antisense-Induced Exon Skipping in Cultured Muscle Cells from Six Different DMD Patients. Hum. Mol. Genet..

[76] Aartsma-Rus A., Janson A.A.M., Kaman W.E., Bremmer-Bout M., Van Ommen G.-J.B., Den Dunnen J.T., Van Deutekom J.C.T. (2004). Antisense-Induced Multiexon Skipping for Duchenne Muscular Dystrophy Makes More Sense. Am. J. Hum. Genet..

[77] Wilton S.D., Lloyd F., Carville K., Fletcher S., Honeyman K., Agrawal S., Kole R. (1999). Specific Removal of the Nonsense Mutation from the Mdx Dystrophin MRNA Using Antisense Oligonucleotides. Neuromuscul. Disord..

[78] Mendell J.R., Rodino-Klapac L.R., Sahenk Z., Roush K., Bird L., Lowes L.P., Alfano L., Gomez A.M., Lewis S., Kota J. (2013). Eteplirsen for the Treatment of Duchenne Muscular Dystrophy. Ann. Neurol..

[79] Harper A.D., Topaloglu H., Mercuri E., Suslov V., Wu L., Ayanoglu C.Y., Tansey M., Previtera M.L., Crozier R.A., Magnus L. (2024). Safety and Efficacy of Viltolarsen in Ambulatory and Nonambulatory Males with Duchenne Muscular Dystrophy. Sci. Rep..

[80] Assefa M., Gepfert A., Zaheer M., Hum J.M., Skinner B.W. (2024). Casimersen (AMONDYS 45^TM^): An Antisense Oligonucleotide for Duchenne Muscular Dystrophy. Biomedicines.

[81] Wagner K.R., Kuntz N.L., Koenig E., East L., Upadhyay S., Han B., Shieh P.B. (2021). Safety, Tolerability, and Pharmacokinetics of Casimersen in Patients with Duchenne Muscular Dystrophy Amenable to Exon 45 Skipping: A Randomized, Double-Blind, Placebo-Controlled, Dose-Titration Trial. Muscle Nerve.

[82] Torres-Masjoan L., Aguti S., Zhou H., Muntoni F. (2025). Clinical Applications of Exon-Skipping Antisense Oligonucleotides in Neuromuscular Diseases. Mol. Ther..

[83] Arechavala-Gomeza V., López-Martínez A., Aartsma-Rus A. (2026). Antisense RNA Therapies for Muscular Dystrophies. J. Neuromuscul. Dis..

[84] Clemens P.R., Rao V.K., Connolly A.M., Harper A.D., Mah J.K., Smith E.C., McDonald C.M., Zaidman C.M., Morgenroth L.P., Osaki H. (2020). Safety, Tolerability, and Efficacy of Viltolarsen in Boys with Duchenne Muscular Dystrophy Amenable to Exon 53 Skipping: A Phase 2 Randomized Clinical Trial. JAMA Neurol..

[85] Wang B., Cao J., Wu J., Zhao Y., Zhang Y., Abendroth F., Lin C., Zhong L., Yu H., Seow Y. (2026). Cardiac and Skeletal Muscle Delivery of Biotherapeutics with a Blood Vessel Epicardial Substance-Targeting Peptide. Biomaterials.

[86] Komaki H., Takeshita E., Kunitake K., Ishizuka T., Shimizu-Motohashi Y., Ishiyama A., Sasaki M., Yonee C., Maruyama S., Hida E. (2025). Phase 1/2 Trial of Brogidirsen: Dual-Targeting Antisense Oligonucleotides for Exon 44 Skipping in Duchenne Muscular Dystrophy. Cell Rep. Med..

[87] Gan L., Wu L.C.L., Wood J.A., Yao M., Treleaven C.M., Estrella N.L., Wentworth B.M., Hanson G.J., Passini M.A. (2022). A Cell-Penetrating Peptide Enhances Delivery and Efficacy of Phosphorodiamidate Morpholino Oligomers in Mdx Mice. Mol. Ther. Nucleic Acids.

[88] Shah M.N.A., Wilton-Clark H., Haque F., Powell B., Sutanto L.E., Maradiya R., Zhabyeyev P., Roshmi R.R., Anwar S., Aslesh T. (2025). DG9 Boosts PMO Nuclear Uptake and Exon Skipping to Restore Dystrophic Muscle and Cardiac Function. Nat. Commun..

[89] Haque U.S., Yokota T. (2023). Enhancing Antisense Oligonucleotide-Based Therapeutic Delivery with DG9, a Versatile Cell-Penetrating Peptide. Cells.

[90] Finkel R.S. (2010). Read-through Strategies for Suppression of Nonsense Mutations in Duchenne/Becker Muscular Dystrophy: Aminoglycosides and Ataluren (PTC124). J. Child Neurol..

[91] Chen G., Katrekar D., Mali P. (2019). RNA-Guided Adenosine Deaminases: Advances and Challenges for Therapeutic RNA Editing. Biochemistry.

[92] Byrne S.M., Burleigh S.M., Fragoza R., Jiang Y., Savva Y., Pabon R., Kania E., Rainaldi J., Portell A., Mali P. (2025). An Engineered U7 Small Nuclear RNA Scaffold Greatly Increases ADAR-Mediated Programmable RNA Base Editing. Nat. Commun..

[93] Goyenvalle A., Vulin A., Fougerousse F., Leturcq F., Kaplan J.C., Garcia L., Danos O. (2004). Rescue of Dystrophic Muscle through U7 SnRNA-Mediated Exon Skipping. Science.

[94] Liao Q., Zhang Y., He J., Huang K. (2022). Global Prevalence of Myotonic Dystrophy: An Updated Systematic Review and Meta-Analysis. Neuroepidemiology.

[95] Takahashi M.P. (2025). Update on the Clinical and Therapeutic Aspects of Myotonic Dystrophy Type 1. Curr. Opin. Neurol..

[96] Nezu Y., Kino Y., Sasagawa N., Nishino I., Ishiura S. (2007). Expression of MBNL and CELF MRNA Transcripts in Muscles with Myotonic Dystrophy. Neuromuscul. Disord..

[97] Piasecka A., Szcześniak M.W., Sekrecki M., Kajdasz A., Sznajder Ł.J., Baud A., Sobczak K. (2024). MBNL Splicing Factors Regulate the Microtranscriptome of Skeletal Muscles. Nucleic Acids Res..

[98] la Fontaine L.A., Bruijnes J.E., Smulders F.H., Gorissen-Brouwers C., Karnebeek I.E., Braakman H.M., Klinkenberg S., Mul K., ‘t Hoen P.B.A., van Kuijk S.M. (2024). Comprehensive Four-Year Disease Progression Assessment of Myotonic Dystrophy Type 1. Neuromuscul. Disord..

[99] Ginjupalli V.K.M., Reisqs J.B., Cupelli M., Chahine M., Boutjdir M. (2025). Cardiac Involvement in Myotonic Dystrophy Type 1: Mechanisms, Clinical Perspectives, and Emerging Therapeutic Strategies. Int. J. Mol. Sci..

[100] Thornton C.A., Moxley R.T., Eichinger K., Heatwole C., Mignon L., Arnold W.D., Ashizawa T., Day J.W., Dent G., Tanner M.K. (2023). Antisense Oligonucleotide Targeting DMPK in Patients with Myotonic Dystrophy Type 1: A Multicentre, Randomised, Dose-Escalation, Placebo-Controlled, Phase 1/2a Trial. Lancet Neurol..

[101] Wheeler T.M., Leger A.J., Pandey S.K., Mac Leod A.R., Wheeler T.M., Cheng S.H., Wentworth B.M., Bennett C.F., Thornton C.A. (2012). Targeting Nuclear RNA for in Vivo Correction of Myotonic Dystrophy. Nature.

[102] Raaijmakers R.H.L., Ripken L., Ausems C.R.M., Wansink D.G. (2019). CRISPR/Cas Applications in Myotonic Dystrophy: Expanding Opportunities. Int. J. Mol. Sci..

[103] van Agtmaal E.L., André L.M., Willemse M., Cumming S.A., van Kessel I.D.G., van den Broek W.J.A.A., Gourdon G., Furling D., Mouly V., Monckton D.G. (2017). CRISPR/Cas9-Induced (CTG⋅CAG)n Repeat Instability in the Myotonic Dystrophy Type 1 Locus: Implications for Therapeutic Genome Editing. Mol. Ther..

[104] Izzo M., Battistini J., Golini E., Voellenkle C., Provenzano C., Orsini T., Strimpakos G., Scavizzi F., Raspa M., Baci D. (2025). Muscle-specific Gene Editing Improves Molecular and Phenotypic Defects in a Mouse Model of Myotonic Dystrophy Type 1. Clin. Transl. Med..

[105] Porquet F., Weidong L., Jehasse K., Gazon H., Kondili M., Blacher S., Massotte L., Di Valentin E., Furling D., Gillet N.A. (2023). Specific DMPK-Promoter Targeting by CRISPRi Reverses Myotonic Dystrophy Type 1-Associated Defects in Patient Muscle Cells. Mol. Ther. Nucleic Acids.

[106] Laurent M., Geoffroy M., Pavani G., Guiraud S. (2024). CRISPR-Based Gene Therapies: From Preclinical to Clinical Treatments. Cells.

[107] Philippou S., Mastroyiannopoulos N.P., Makrides N., Lederer C.W., Kleanthous M., Phylactou L.A. (2018). Selection and Identification of Skeletal-Muscle-Targeted RNA Aptamers. Mol. Ther. Nucleic Acids.

[108] Li X., Xu J., Yao S., Zhang N., Zhang B.T., Zhang Z.K. (2025). Targeting Drug Delivery System to Skeletal Muscles: A Comprehensive Review of Different Approaches. J. Cachexia Sarcopenia Muscle.

[109] Malecova B., Sala D., Melikian G.M., Johns R., Erdogan G., Hartmann M., Jordan M., Arias J.D., Bhattacharya A., Meng Q. (2026). Development of a DUX4-Targeting Antibody Oligonucleotide Conjugate as a Therapy for FSHD. Nucleic Acids Res..

[110] Geng L.N., Yao Z., Snider L., Fong A.P., Cech J.N., Young J.M., vanderMaarel S.M., Ruzzo W.L., Gentleman R.C., Tawil R. (2012). DUX4 Activates Germline Genes, Retroelements, and Immune Mediators: Implications for Facioscapulohumeral Dystrophy. Dev. Cell.

[111] Lemmers R.J.L.F., Van Der Vliet P.J., Klooster R., Sacconi S., Camaño P., Dauwerse J.G., Snider L., Straasheijm K.R., Van Ommen G.J., Padberg G.W. (2010). A Unifying Genetic Model for Facioscapulohumeral Muscular Dystrophy. Science.

[112] Statland J., Tawil R. (2014). Facioscapulohumeral Muscular Dystrophy. Neurol. Clin..

[113] Chen T.H., Wu Y.Z., Tseng Y.H. (2020). Early-Onset Infantile Facioscapulohumeral Muscular Dystrophy: A Timely Review. Int. J. Mol. Sci..

[114] Zhang A., Lim K.R.Q., Chen Z., Yokota T., Chen Y.-W. (2026). DUX4 Reduction and Muscle Function Improvement by Subcutaneous Delivery of Gapmer Antisense Oligonucleotides. Mol. Ther. Nucleic Acids.

[115] Bouwman L.F., den Hamer B., van den Heuvel A., Franken M., Jackson M., Dwyer C.A., Tapscott S.J., Rigo F., van der Maarel S.M., de Greef J.C. (2021). Systemic Delivery of a DUX4-Targeting Antisense Oligonucleotide to Treat Facioscapulohumeral Muscular Dystrophy. Mol. Ther. Nucleic Acids.

[116] Lu-Nguyen N., Malerba A., Herath S., Dickson G., Popplewell L. (2021). Systemic Antisense Therapeutics Inhibiting DUX4 Expression Ameliorates FSHD-like Pathology in an FSHD Mouse Model. Hum. Mol. Genet..

[117] Lek A., Atas E., Lin B., Hesterlee S.E., Bönnemann C.G., Byrne B.J. (2026). Meeting Report: 2024 Muscular Dystrophy Association Summit on ‘Safety and Challenges in Gene Therapy of Neuromuscular Diseases’. J. Neuromuscul. Dis..

[118] Makkar S.K. (2025). Advances in RNA-Based Therapeutics: Current Breakthroughs, Clinical Translation, and Future Perspectives. Front. Genet..

[119] McGreevy J.W., Hakim C.H., McIntosh M.A., Duan D. (2015). Animal Models of Duchenne Muscular Dystrophy: From Basic Mechanisms to Gene Therapy. DMM Dis. Models Mech..

[120] Canonico F., Chirivi M., Maiullari F., Milan M., Rizzi R., Arcudi A., Galli M., Pane M., Gowran A., Pompilio G. (2022). Focus on the Road to Modelling Cardiomyopathy in Muscular Dystrophy. Cardiovasc. Res..

[121] Partridge T.A. (2013). The Mdx Mouse Model as a Surrogate for Duchenne Muscular Dystrophy. FEBS J..

[122] Kornegay J.N., Bogan J.R., Bogan D.J., Childers M.K., Li J., Nghiem P., Detwiler D.A., Larsen C.A., Grange R.W., Bhavaraju-Sanka R.K. (2012). Canine Models of Duchenne Muscular Dystrophy and Their Use in Therapeutic Strategies. Mamm. Genome.

[123] Nakamura A., Takeda S. (2011). Mammalian Models of Duchenne Muscular Dystrophy: Pathological Characteristics and Therapeutic Applications. J. Biomed. Biotechnol..

[124] Otake M., Imamura M., Enya S., Kangawa A., Shibata M., Ozaki K., Kimura K., Ono E., Aoki Y. (2024). Severe Cardiac and Skeletal Manifestations in DMD-Edited Microminipigs: An Advanced Surrogate for Duchenne Muscular Dystrophy. Commun. Biol..

[125] Echigoya Y., Trieu N., Duddy W., Moulton H.M., Yin H., Partridge T.A., Hoffman E.P., Kornegay J.N., Rohret F.A., Rogers C.S. (2021). A Dystrophin Exon-52 Deleted Miniature Pig Model of Duchenne Muscular Dystrophy and Evaluation of Exon Skipping. Int. J. Mol. Sci..

[126] Jafar-Nejad P., Powers B., Soriano A., Zhao H., Norris D.A., Matson J., Debrosse-Serra B., Watson J., Narayanan P., Chun S.J. (2021). The Atlas of RNase H Antisense Oligonucleotide Distribution and Activity in the CNS of Rodents and Non-Human Primates Following Central Administration. Nucleic Acids Res..

[127] Kondratov O., Kondratova L., Mandel R.J., Coleman K., Savage M.A., Gray-Edwards H.L., Ness T.J., Rodriguez-Lebron E., Bell R.D., Rabinowitz J. (2021). A Comprehensive Study of a 29-Capsid AAV Library in a Non-Human Primate Central Nervous System. Mol. Ther..

[128] Molinaro M., Torrente Y., Villa C., Farini A. (2024). Advancing Biomarker Discovery and Therapeutic Targets in Duchenne Muscular Dystrophy: A Comprehensive Review. Int. J. Mol. Sci..

[129] Coenen-Stass A.M.L., Sork H., Gatto S., Godfrey C., Bhomra A., Krjutškov K., Hart J.R., Westholm J.O., O’Donovan L., Roos A. (2018). Comprehensive RNA-Sequencing Analysis in Serum and Muscle Reveals Novel Small RNA Signatures with Biomarker Potential for DMD. Mol. Ther. Nucleic Acids.

[130] Fortunato F., Ferlini A. (2023). Biomarkers in Duchenne Muscular Dystrophy: Current Status and Future Directions. J. Neuromuscul. Dis..

[131] Anthony K., Arechavala-Gomeza V., Taylor L.E., Vulin A., Kaminoh Y., Torelli S., Feng L., Janghra N., Bonne G., Beuvin M. (2014). Dystrophin Quantification Biological and Translational Research Implications. Neurology.

[132] Siracusa J., Koulmann N., Banzet S. (2018). Circulating MyomiRs: A New Class of Biomarkers to Monitor Skeletal Muscle in Physiology and Medicine. J. Cachexia Sarcopenia Muscle.

[133] Andersen G., Dahlqvist J.R., Vissing C.R., Heje K., Thomsen C., Vissing J. (2017). MRI as Outcome Measure in Facioscapulohumeral Muscular Dystrophy: 1-Year Follow-up of 45 Patients. J. Neurol..

[134] Yoon D.Y., Daniels M.J., Willcocks R.J., Triplett W.T., Morales J.F., Walter G.A., Rooney W.D., Vandenborne K., Kim S. (2024). Five Multivariate Duchenne Muscular Dystrophy Progression Models Bridging Six-Minute Walk Distance and MRI Relaxometry of Leg Muscles. J. Pharmacokinet. Pharmacodyn..

[135] Mcdonald C.M., Henricson E.K., Abresch R.T., Florence J., Eagle M., Gappmaier E., Glanzman A.M., Spiegel R., Barth J., Elfring G. (2013). The 6-Minute Walk Test and Other Clinical Endpoints in Duchenne Muscular Dystrophy: Reliability, Concurrent Validity, and Minimal Clinically Important Differences from a Multicenter Study. Muscle Nerve.

[136] Han J.J., De Bie E., Nicorici A., Abresch R.T., Bajcsy R., Kurillo G. (2015). Reachable Workspace Reflects Dynamometer-Measured Upper Extremity Strength in Facioscapulohumeral Muscular Dystrophy. Muscle Nerve.

[137] Heatwole C., Bode R., Johnson N.E., Dekdebrun J., Dilek N., Eichinger K., Hilbert J.E., Logigian E., Luebbe E., Martens W. (2016). Myotonic Dystrophy Health Index: Correlations with Clinical Tests and Patient Function. Muscle Nerve.

[138] Main M., Kairon H., Mercuri E., Muntoni F. (2003). The Hammersmith Functional Motor Scale for Children with Spinal Muscular Atrophy: A Scale to Test Ability and Monitor Progress in Children with Limited Ambulation. Eur. J. Paediatr. Neurol..

[139] Alhamadani F., Zhang K., Parikh R., Wu H., Rasmussen T.P., Bahal R., Zhong X.B., Manautou J.E. (2022). Adverse Drug Reactions and Toxicity of the Food and Drug Administration–Approved Antisense Oligonucleotide Drugs. Drug Metab. Dispos..

[140] Jackson A.L., Bartz S.R., Schelter J., Kobayashi S.V., Burchard J., Mao M., Li B., Cavet G., Linsley P.S. (2003). Expression Profiling Reveals Off-Target Gene Regulation by RNAi. Nat. Biotechnol..

[141] Ranjbar S., Zhong X., Manautou J., Lu X. (2023). A Holistic Analysis of the Intrinsic and Delivery-Mediated Toxicity of SiRNA Therapeutics. Adv. Drug Deliv. Rev..

[142] Judge A.D., Bola G., Lee A.C.H., MacLachlan I. (2006). Design of Noninflammatory Synthetic SiRNA Mediating Potent Gene Silencing in Vivo. Mol. Ther..

[143] Zhang Y., Engelman J., Friedlander R.M. (2009). Allele-Specific Silencing of Mutant Huntington’s Disease Gene. J. Neurochem..

[144] Singh A., Tekade M., Nagaraja S., Bharti A., Tekade R.K. (2026). Advancements in RNA-Based Therapies from Bench to Bedside. npj Drug Discov..

[145] Hammond S.M., Aartsma-Rus A., Alves S., Borgos S.E., Buijsen R.A.M., Collin R.W.J., Covello G., Denti M.A., Desviat L.R., Echevarría L. (2021). Delivery of Oligonucleotide-based Therapeutics: Challenges and Opportunities. EMBO Mol. Med..

[146] Michel S., Schirduan K., Shen Y., Klar R., Tost J., Jaschinski F. (2021). Using RNA-Seq to Assess Off-Target Effects of Antisense Oligonucleotides in Human Cell Lines. Mol. Diagn. Ther..

[147] Ruan H., Dou D., Lu J., Xiao X., Gong X., Zhang X. (2025). Off-Target Effects of Oligonucleotides and Approaches of Preclinical Assessments. SLAS Discov..

[148] Lemaitre M.M. (2022). Individualized Antisense Oligonucleotide Therapies: How to Approach the Challenge of Manufacturing These Oligos from a Chemistry, Manufacturing, and Control-Regulatory Standpoint. Nucleic Acid Ther..

